# Turpentine in Typhoid

**Published:** 1892-08

**Authors:** J. J. Waller

**Affiliations:** Oliver Springs, Tenn.


### TURPENTINE IN TYPHOID.

BY J. J. WALLER, M. D., OLIVER SPRINGS, TENN.

It is an extensive practice and almost an universal rule to treat typhoid, so far as medication is concerned, on the antiseptic and antipyretic plan; and in regard to the antiseptic used, turpentine seems to stand at the head of the list. Many others, of course, have their advocates. The condition of affairs and array of symptoms in a regular case of typhoid seems to be peculiarly met by the physiological action of turpentine.

It is hardly probable that turpentine passes down the alimentary tract, as is generally supposed, and acts locally on the inflamed Peyer's patches, for it is well known that the stimulating effect of the turpentine is felt and realized long before the agent has time to travel several feet down the intestine and reach the seat of the lesions. It is probable, and even likely, that a small portion of it may do so, but the amount must necessarily be so small that the good effect from the turpentine is brought about in a different way.

I do not believe that typhoid should be considered to dwell alone in Peyer's patches; the nervous system is prostrated, especially the sympathetic. The secretions, and they are governed by the sympathetic, do not continue with the usual vigor. The mouth is dry, stomach often nauseated and the bowels tympanitic and full of gas—all we imagine due to the lack of proper secretion.

Turpentine is a good stimulant to the sympathetic, for under its use the mouth becomes moist and the tympanites relieved. If the secretions of the intestines, both mucous and serous, be aroused by turpentine (which we assume to be a fact), the gentle vermicular action is facilitated, and as a result the gases are either not generated, or else they are expelled or absorbed, and the pliant coat of the bowel is not so subject to extensive ulceration and inflammation.

The proper amount of turpentine in any case has to be determined by the effect it has in that particular case, for the state of the secretions is the guide to the amount used. It is well enough to bear in mind that turpentine sometimes deals harshly with the kidneys and brain, and in some instances it seems to be poisonous to the whole system, and cannot be borne at all. When such is the case, its bad effects may be overcome by combining it with other suitable remedies. For example, the following will illustrate :

R Spirits Turpentine .......................... dr. iss

Po. Acaciæ ..............................

Sach. Albæ .............................. aa dr. ii

Tr. Opii Camph ..............................

Listerine ..............................

Aq. Camphoræ ..............................

Aq. Cinnamomi .............................. aa oz. ii

M. Ft. Emulsion.

The opium, benzoic acid, ol. anise and camphor protect the kidneys, and at the same time the opium and camphor support and quiet the brain and nervous system. The listerine keeps the stomach in good condition, while the eucalypus it contains acts as a febrifuge tonic.

With turpentine as the chief, and the other named remedies to corroborate and correct, I find to be good medication in typhoid.

